# A cluster randomised trial of the program to enhance adjustment to residential living (PEARL): a novel psychological intervention to reduce depression in newly admitted aged care residents

**DOI:** 10.1186/s12877-020-1492-5

**Published:** 2020-03-12

**Authors:** Tanya E. Davison, Marita P. McCabe, Lucy Busija, Daniel W. O’Connor, Vera Camões Costa, Jessica Byers

**Affiliations:** 1 Health and Ageing Research Group, School of Health Sciences, Faculty of Health, Arts and Design, Swinburne University of Technology, H95 PO Box 218, Hawthorn, VIC 3122 Australia; 2 Biostatistics Consulting Platform, Research Methodology Division, Department of Epidemiology and Preventive Medicine, School of Public Health and Preventative Medicine, Monash University, Melbourne, Australia; 3 Department of Health and Human Services, Melbourne, VIC Australia

**Keywords:** Residential aged care, Long term care, Nursing homes, Adjustment, Depression, Quality of life

## Abstract

**Background:**

Depression rates are high in residential aged care (RAC) facilities, with newly admitted residents at particular risk. New approaches to address depression in this population are urgently required, particularly psychological interventions suitable for widespread use across the RAC sector. The Program to Enhance Adjustment to Residential Living (PEARL) is a brief intervention, designed to provide individually tailored care approaches to meet the psychological needs of newly admitted residents, delivered in collaboration with facility staff.

**Methods:**

PEARL will be evaluated using a cluster randomised controlled design, comparing outcomes for residents who participate in the intervention with those residing in care as usual control facilities. Participants are RAC residents aged 60 years or above, with normal cognition or mild-moderate cognitive impairment, who relocated to the facility within the previous 4 weeks. The primary outcomes are depressive symptoms and disorders, with secondary outcomes including anxiety, stress, quality of life, adjustment to RAC, and functional dependence, analysed on an intention to treat basis using multilevel modelling.

**Discussion:**

PEARL is an intervention based on self-determination theory, designed to reduce depression in newly admitted residents by tailoring day to day care to meet their psychological needs. This simple psychological approach offers an alternative care model to the current over-reliance of antidepressant medications.

**Trial registration:**

ACTRN12616001726448; Registered 16 December 2016 with the Australian New Zealand Clinical Trials Registry.

## Background

Late life depression is associated with higher mortality and increased care needs [1], with the burden of this illness set to worsen as the population ages. Depression is particularly common in residential aged care (RAC) settings, with Australian data indicating that approximately one-half of residents live with depressive symptoms [2], while the median prevalence estimate of Major Depressive Disorder (MDD) in an international review of studies was 10% [3]. Current treatment approaches consist almost entirely of antidepressant medications, despite the failure of literature reviews to clearly establish the effectiveness of antidepressants in RAC settings [4], and concerns raised about their low efficacy [5, 6] and adverse effects [5] in older people with dementia in particular.

There is preliminary evidence that psychological interventions are helpful for depression in RAC [7], although there are few high-quality trials in the existing literature. The field lacks well-validated interventions designed specifically for this elderly, frail and clinically complex population. Many existing psychotherapies, such as cognitive behavioural therapy, rely on skilled mental health clinicians, and so are not feasible for widespread use in Australian facilities, where there has traditionally been limited access to specialist psychological services [8]. New approaches are required to address this problem.

Researchers and clinicians have so far failed to appreciate the significance of the transition period for mental health. On admission to a RAC facility, older people typically find themselves thrust into an unfamiliar environment, often at a time of crisis, after an acute medical event, hospitalisation, or following the death of a family caregiver. In many cases, the older adult is admitted against their wishes, with associated family conflict, and without time to adequately prepare for this major life event. Many residents fail to adjust successfully to their changed circumstances, even several months after the relocation [9]. Depression is often present at the point of entry to care [10] and new cases commonly arise throughout the transition period [11]. Thus, the transition period provides an ideal opportunity to address pre-existing depressive symptoms and prevent this high-risk population from developing new conditions.

We have developed a novel psychological approach for older people newly admitted to RAC: The Program to Enhance Adjustment to Residential Living (PEARL). This program aims to mitigate the risks of depression and help older adults to adjust more successfully to institutional care through tailored care approaches that address their key psychological needs. This program is based on self-determination theory, which posits that psychological health throughout the lifespan is dependent on three basic needs being satisfied: competence, autonomy and relatedness [12]. The relationship between satisfaction of these basic needs and wellbeing has been confirmed in research in RAC settings [13], with additional evidence that depression is associated with low levels of autonomy and environmental mastery (a construct related to competence) [14]. There is a large literature documenting low levels of autonomy and poor social relations among people living in RAC, which are reportedly associated with poor adjustment to the new setting [9, 15].

To date, there has been no research testing the impact of an intervention designed to address older people’s needs for competence, autonomy and relatedness on their wellbeing and mental health, despite the indications that this approach could be beneficial. This study aims to address the gap in knowledge of how to effectively target high rates of depression in RAC settings, through a controlled trial of an individually tailored care approach addressing psychological needs of newly admitted residents.

### Hypotheses and expected outcomes

It is hypothesised that PEARL plus usual care (‘intervention condition’) is superior to usual care alone (‘control condition’) in reducing depression in newly admitted residents, including reduction in the level of depressive symptoms and in the likelihood of meeting DSM-5 criteria for MDD. It is also hypothesised that the intervention will improve residents’ anxiety, quality of life and perceived adjustment to the RAC facility, compared to usual care alone.

## Methods/design

### Study design

The efficacy of PEARL in reducing MDD and symptoms of depression in RAC residents is evaluated with a cluster randomised controlled trial design. Randomisation is applied at the facility level to avoid contamination of the intervention and control conditions by the close involvement of RAC facility staff in the intervention.

### Sample population and recruitment procedure

RAC facilities (known internationally as nursing homes, assisted living facilities, or long-term care facilities) across metropolitan Melbourne, Australia with a minimum of 50 resident places are being approached to participate in the study. Facilities are randomly allocated to the intervention or control condition by a biostatistician not otherwise involved in delivery of the intervention or data collection. Randomisation of facilities is conducted using a web-based random allocation sequence generator (https://www.random.org/) and stratified by organisation and number of resident places (< 100 vs 100+), to ensure that each condition contains a spread of different organisations and facility sizes, with these organisational factors considered potential contributors to study outcomes. Facilities are randomised upon enrolment into the study, before data collection begins.

Participating facilities are informed of the condition to which their facility has been allocated and asked to nominate residents who are potentially suitable to participate in the trial. Inclusion criteria are (i) aged 60 years or above; and (ii) admitted to the RAC facility within the previous 4 weeks. Exclusion criteria are (i) acute severe medical illness likely to compromise participation in the program; (ii) moderate-severe cognitive impairment, operationalised as a Mini-Mental State Examination (MMSE) score of less than 15 [16]; and (iii) non-fluency in English. Residents at all levels of depression, as well as those without symptoms of depression, are eligible to participate.

Facility staff are asked to identify residents who meet the inclusion criteria and who have an absence of severe cognitive impairment and notify the research team. These residents are then are approached by a researcher for consent and eligibility screening, including administration of the MMSE [16]. Consent is obtained from the participant, as well as by their next-of-kin in cases where the MMSE score is less than 24. Participants are blind to their study condition until after they have consented. Research assistants collecting outcome data are also blind to study condition, with a process in place to record instances where they become unblinded, for example, through disclosure by the participant or facility staff.

**Fig. 1:**
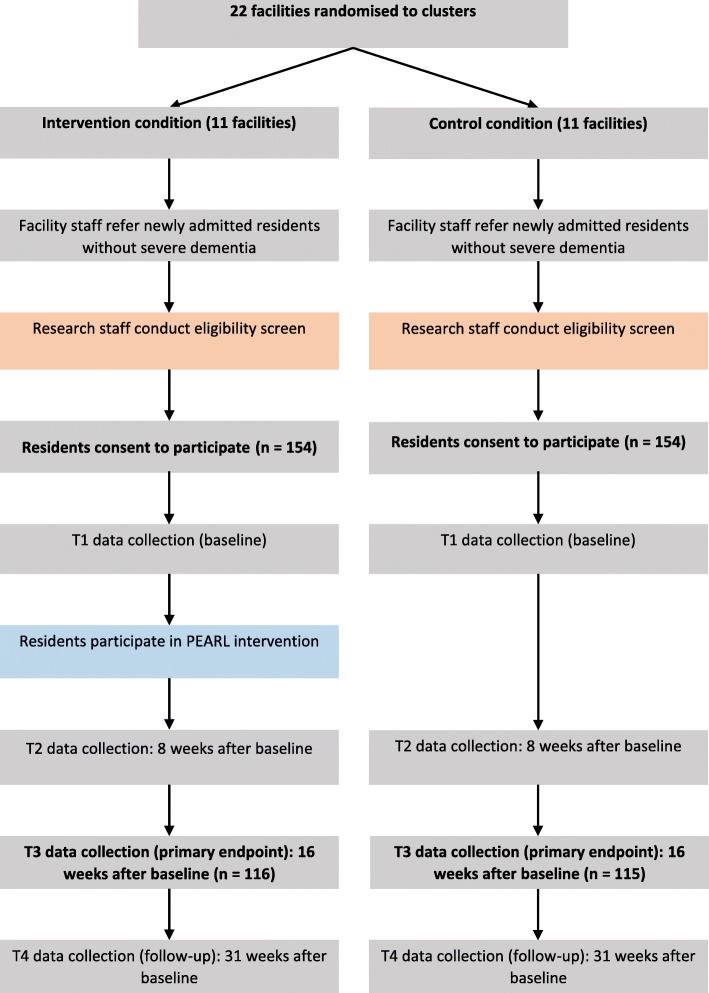
CONSORT flow diagram

We aim to recruit 308 participants from 22 RAC facilities. Additional facilities will be recruited if the target sample size is not reached working with 22 facilities. Estimations were based on expectations of *k* = 22 facilities, with an average of 14 participants each (*n* = 308), adjusted to allow for: (i) a within-cluster correlation, reflected in an intra-class correlation of .054, and (ii) attrition rate of 25% over 6 months, based on previous Australian longitudinal samples in RAC [17, 18]. The final sample of *n* = 231 participants at the primary endpoint (6 months post-baseline) will provide 80% power to detect a moderate effect in depressive symptoms (d = .48) [19], with 5% Type I error (two-sided). The moderately small effect size approximately corresponds to r-square of 0.05 (i.e., the intervention explains at least 5% of variance in depressive symptoms). The selected effect size was based on meta-analyses of psychological interventions for depression with older adults, which reported overall mean effect sizes of g = 0.64 [20] and g = 0.57 [7]. Figure 1 is a CONSORT flow diagram of participants in the trial.

### Intervention

PEARL is a brief, simple, individually tailored intervention that is delivered over 7 weeks using a structured manual. It is designed to be suitable for use with residents of normal cognition or mild to moderate dementia newly admitted to a RAC facility, with strategies employed tailored to each resident’s needs, interests, aptitudes and background. In recognition of the difficulty accessing specialist mental health clinicians in RAC settings, PEARL is designed to be administered by a range of trained personnel. However, to minimise concerns about treatment fidelity in this efficacy trial, it is being delivered by trained mental health clinicians. PEARL is based on self-determination theory [12], and the clinician collaborates with residents and facility staff to develop care approaches that enhance residents’ autonomy, competence, and social relations. Involvement of facility staff in psychological interventions has previously been suggested to improve outcomes for residents with depression [7].

The program comprises three weekly 45- to 60-min individual sessions with residents in their own rooms. Two additional ‘booster’ sessions are provided 2 and 4 weeks after the final session to review and modify strategies, with a key facility staff member joining these sessions. The clinician meets with a key facility staff member (typically a nurse, care assistant, or diversional therapist) after each resident session to discuss care approaches that will better meet the residents’ individual psychological needs, and how these approaches can be feasibly implemented in the facility. Clinical record forms, documenting the key outcomes of each session, including agreed strategies to implement, are completed in collaboration with the resident, refined with the staff member following the session, and filed in the resident’s file at the facility. Two follow-up telephone consultations are conducted with the key facility staff member 2 and 4 weeks after the final resident session, to encourage and support staff in continuing to employ the recommended strategies and general therapeutic approach.

The content of the PEARL program was finalised in consultation with an advisory committee of senior RAC managers, to ensure feasibility for the RAC setting. Detailed information about the content of the program is in Table 1.

**Table 1:** Content of the PEARL intervention

Session	Content	Participants
Session 1	Validation, orientation and social relations	Resident
• Introduction to PEARL • Validating and normalising the resident’s experience of relocating to the facility • Orientation to the facility and broader community • Relationships with family and friends • Forming relationships with other residents		
Session 2	Increasing meaningful activity and enhancing competence	Resident
• Understanding the resident’s previous activities, occupation, skills, and interests • Assessing preferences for day to day activities, using a structured tool to determine those that enhance competence • Developing an implementation plan to increase meaningful activity		
Session 3	Enhancing autonomy	Resident
• Assessing preferences for level of choice, independence and autonomy in their environment and day to day activities, including care tasks, using a structured tool • Developing a plan to increase autonomy		
Booster 1	Review and problem-solving	Resident and staff member
• Introducing the role of the staff member in supporting the resident in meeting their goals • Reviewing the program, individual strategies selected for implementation, and the resident’s progress in meeting goals • Reinforcing successes and problem-solve solutions to address barriers, using a problem-solving approach and refining strategies if required		
Booster 2	Review and problem-solving, and future planning	Resident and staff member
• Reviewing the program, reinforcing resident’s successes and achievements. • Providing assistance in problem-solving any difficulties reported by resident. • Preparing for the future: How to apply the PEARL approach to deal with future issues that emerge.		

Clinicians delivering the program have previous experience in delivering psychological treatments and receive training and regular supervision in implementing the intervention. Treatment fidelity is monitored using a review of audiotaped sessions, with sessions coded for adherence to the intervention manual. The review will be undertaken on 5% of sessions randomly selected from all sessions.

Participants in the control group do not receive any additional intervention beyond the usual care offered by the facility. This includes group leisure activities, assistance with care, and medications, which are provided to participants in both intervention and control groups.

### Outcomes

Data will be collected from the intervention and control participants at baseline (within 4 weeks of admission to the RAC facility, T1), 8 weeks after baseline (T2), 16 weeks after baseline (T3), and 31 weeks after baseline (T4) (see Table 2 for participant timeline). The primary endpoint is T3, which is 2 months post-intervention. The T4 data collection point equates to a six-month post-intervention follow-up.

**Table 2:** Participant timeline

TIMEPOINT	Enrolment	T1	Intervention period	T2	T3	T4
	1–4 weeks prior to baseline	Baseline	Week 1, 2, 3, 5, 7	Week 8	Weeks 16	Week 31
Enrolment:						
Eligibility screen	X					
Informed consent	X					
Interventions:						
PEARL – 3 sessions, 2 boosters			X			
Care as usual control			X			
Assessments:						
Mini-Mental Status Examination	X					X
Cornell Scale		X		X	X	X
SCID-5-CD – Major depressive disorder		X		X	X	X
Geriatric Anxiety Scale		X		X	X	X
Quality of Life in Alzheimer’s Disease		X		X	X	X
DASS-21 – Stress scale		X		X	X	X
Index of Relocation Adjustment Scale		X		X	X	X
Instrumental Activities of Daily Living Scale		X		X	X	X
Basic Needs Satisfaction in Life Scale		X		X	X	X
Importance of Basic Needs Scale		X		X	X	X
Meaningful Activity in Residential Care Scale		X		X	X	X
View of Relocation Scale		X				
Medical and psychiatric conditions and treatment		X		X	X	X

*SCID-5-CV* Structured Clinical Interview for DSM-5 Disorders – Clinician Version

*DASS-21* Depression, Anxiety and Stress Scale-21

The primary outcome measures are depressive symptoms and disorders. The presence of current MDD will be determined using the Structured Clinical Interview for DSM-5 Disorders – Clinician Version (SCID-5-CV) [21], modified for administration to both the participant and a staff informant, as recommended previously [22], with additional prompting designed to assist in identifying symptoms of depression in older adults in RAC settings. Depressive symptoms will be assessed using the Cornell Scale for Depression in Dementia [23] on the basis of a resident interview and staff informant interview. The final score on each item represents the administrator’s clinical judgement based on information collected in the interviews, including observations of the participant. The Cornell scale is the most commonly used measure of depressive symptoms in RAC settings, both in routine screening and research studies, and has been validated for participants with and without dementia [24]. Research assistants receive substantial training and ongoing supervision in the use of both the SCID-5-CV and Cornell Scale.

Secondary outcomes include anxiety, stress, quality of life, adjustment to RAC, and functional dependency. Anxiety is assessed using the 20-item self-rated Geriatric Anxiety Inventory [25]. Stress is assessed using the Stress scale of the Depression, Anxiety and Stress Scale-21 [26]. Quality of life is assessed using the Quality of Life in Alzheimer’s Disease [27], modified for use in RAC [28]. This 15-item scale is administered to both the participant and a facility staff informant. Adjustment to RAC is assessed using the Index of Relocation Adjustment scale [29], modified to include an additional item that assesses the degree to which residents feel ‘at home’ in the facility. Functional dependency is assessed using the Instrumental Activities of Daily Living Scale [30], adapted for the RAC setting [14], and completed by a facility staff informant, with assistance from the research assistant, to maximise informant consistency.

Several variables are assessed as potential mechanisms of change (mediators), relating to the targets of the intervention, including satisfaction of basic psychological needs (autonomy, competence and social relations), which is assessed using the 21-item Basic Needs Satisfaction in Life Scale [31]. A 15-item Importance of Basic Needs Scale was developed specifically for this study to assess residents’ perceptions of the importance of these three basic psychological needs. The Meaningful Activity in Residential Care Scale is a 9-item scale designed for this study to assess the degree to which residents feel they have the opportunity to engage in meaningful activity. A scale was also developed to assess residents’ perception of their relocation to RAC: the 12-item View of Relocation Scale, which is assessed at T1 as a potential moderating variable. This scale assesses the degree to which the resident perceives they had control in the decision to relocate to RAC and the degree to which the resident perceives that the relocation was warranted. The three scales constructed for the purposes of the current study have not been previously validated; however, psychometric evaluation of the scales will be reported in subsequent publications reporting trial results. Copies of the scales are available in the supplementary files.

The following resident demographics are recorded at baseline: age, gender, and admission date. Resident cognition is assessed using the Mini Mental State Examination [16] during screening, and also at T4 to account for changes during the study period. Medical and psychiatric history, including pharmacological and non-pharmacological treatments for depression are obtained from the resident’s file at each time point, while the resident will indicate self-rated health at each interview using items 1 and 2 from the Physical Functioning Scale of the Short-Form 36 [32]. Facility characteristics (staff: resident ratio, staff turnover, number of resident places, type of facility) are obtained directly from the participating facility managers. In addition, the Sheltered Care Environment Scale [33] is administered to a random sample of between 4 and 10 staff at each facility as a measure of organisational climate.

### Statistical analysis

Prior to statistical analysis, data entry will be checked through a random sample of baseline assessments that are entered twice; if data entry is found to be problematic (less than 99% concordance rate), double data entry will be employed for all assessment data. In addition, frequencies will be generated for all variables, to ensure valid range values have been entered.

Descriptive statistics will be used to summarise baseline characteristics and pattern of change in participants over time. Primary analyses will be on an intention to treat basis, with supplementary ‘per protocol’ analyses. To account for the within-facility clustering of participants and repeated assessments, differences in the outcomes of the intervention and control groups will be compared with multilevel modelling [34]. For each outcome, a separate three-level model will be specified, with repeated measurements as level 1, individuals as level 2, and RAC facilities as level 3. The models will include group allocation, assessment time, and group by time interaction as predictors, medication use at each assessment time, and organisational climate and facility size < 100/100+ (both measured at the level of a RAC facility) as covariates. Individual and RAC facility variables will be modelled as random effects and the remaining variables will be modelled as fixed effects. Supplementary analyses will test treatment allocation by cognitive impairment interaction to examine whether the effect of intervention differs according to the presence of cognitive impairment. To explore the mediational role of autonomy, competence, and social relations on the association between intervention and depression, multilevel path analysis (with individuals clustered within RACFs) will be undertaken, controlling for organisational climate. In all analyses, missing data will be assumed to be missing at random and will be handled with conditional maximum likelihood estimation. The impact of possible non-random attrition will be explored with simulation analyses [35].

## Discussion

Reducing the substantial burden of depression in RAC settings requires alternative models of care. To date, there is an absence of robust evidence on the use of non-pharmacological approaches to reduce depression in this setting. We propose that the post-admission period provides an opportunity to intervene at a point of high risk of depression, to both address existing symptoms and prevent new conditions from emerging. This project evaluates a simple psychological approach that tailors day to day care to meet the individual psychological needs of newly admitted RAC residents, based on self-determination theory. Individual outcomes will be tracked over the 8 months following admission. While simple, this approach represents a major shift from the current task-focused approach employed in this setting, and, if found to be effective, offers a model that could be widely disseminated across the RAC sector.

## Supplementary information

**Additional file 1.** Scales designed specifically for this study. List of items in three scales were designed specifically for this trial: Meaningful Activity in Residential Care, Importance of Basic Needs, View of Relocation Scale.

**Additional file 2.** SPIRIT 2013 Checklist. Completed the following checklist: Standard Protocol Items: Recommendations for Interventional Trials (SPIRIT) 2013.

## Data Availability

Not applicable.

## Abbreviations

MDD: Major Depressive Disorder
MMSE: Mini-Mental State Examination
PEARL: Program to Enhance Adjustment to Residential Living
RAC: Residential aged care
SCID-5-CV: Structured Clinical Interview for DSM-5 Disorders – Clinician Version.

## References

[1] Mozley C, Sutcliffe C, Bagley H (2004). Toward quality care: outcomes for older people in care homes.

[2] Australian Institute of Health and Welfare (2013). Depression in residential aged care 2008–2012. Aged care statistics series No. 39. Cat. No. AGE 73.

[3] Seitz D, Purandare N, Conn D (2010). Prevalence of psychiatric disorders among older adults in long-term care homes: a systematic review. Int Psychogeriatr.

[4] Boyce RD, Hanlon JT, Karp JF (2012). A review of the effectiveness of antidepressant medications for depressed nursing home residents. J Am Med Dir Assoc.

[5] Farina N, Morrell L, Banerjee S (2017). What is the therapeutic value of antidepressants in dementia? A narrative review. Int J Geriatr Psychiatry.

[6] Nelson JC, Devanand DP (2011). A systematic review and meta-analysis of placebo-controlled antidepressant studies in people with depression and dementia. J Am Geriatr Soc.

[7] Cody RA, Drysdale K (2013). The effects of psychotherapy on reducing depression in residential aged care: a meta-analytic review. Clin Gerontol.

[8] Stargatt J, Bhar S, Davison TE (2016). (2016). The availability of psychological services for aged care residents in Australia: a survey of facility staff. Aust Psychol.

[9] Davison TE, Camões Costa V, Clark A. Adjusting to life in a residential aged care facility: perspectives of people with dementia, family members and facility care staff. J Clin Nurs. 2019;28(21-22):3901–13. 10.1111/jocn.14978 31246319

[10] McSweeney K, O’Connor DW (2008). Depression among newly admitted Australian nursing home residents. Int Psychogeriatr.

[11] Hoover DR, Siegel M, Lucas J (2010). Depression in the first year of stay for elderly long-term nursing home residents in the USA. Int Psychogeriatr.

[12] Ryan RM, Deci EL (2000). Self-determination theory and the facilitation of intrinsic motivation, social development, and well-being. Am Psychol.

[13] Kasser VG, Ryan RM (1999). The relation of psychological needs for autonomy and relatedness to vitality, well-being, and mortality in a nursing home. J App Soc Psychol.

[14] Davison TE, McCabe MP, Knight T, Mellor D (2012). Biopsychosocial factors related to depression in aged care residents. J Affect Disord.

[15] Brownie S, Horstmanshof L, Garbutt R (2014). Factors that impact residents’ transition and psychological adjustment to long-term aged care: a systematic literature review. Int J Nurs Stud.

[16] Folstein MB, Spitzer RL, Gibbon M, Williams JBW (1997). Structured clinical interview for DSM-IV axis I disorders.

[17] Davison TE, Runci S, Eppingstall B, O’Connor DW (2017). A pilot trial of acceptance and commitment therapy for symptoms of depression and anxiety in older adults residing in long term care facilities. Aging Ment Health.

[18] McCabe MP, Bird M, Davison TE (2015). An RCT to evaluate the utility of a clinical protocol for staff in the management of behavioral and psychological symptoms of dementia in residential aged-care settings. Aging Ment Health.

[19] Cohen J (1992). A power primer. Psych Bull.

[20] Cuijpers P, Karyotaki E, Pot AM, Park M, Reynolds CF (2014). Managing depression in older age: psychological interventions. Maturitas.

[21] First MB, Williams JBW, Karg RS, Spitzer RL (2016). Structured clinical interview for DSM-5 disorders – clinician version (SCID-5-CV).

[22] Davison TE, McCabe MP, Mellor D (2009). (2009). An examination of the ‘gold standard’ diagnosis of major depression in research with aged care residents. Am J Geriatr Psychiatry.

[23] Alexopoulos GS, Abrams RC, Young R (1992). Cornell scale for depression in dementia. Biol Psychiatry.

[24] McCabe MP, Davison TE, Mellor D (2006). Depression among older people with cognitive impairment: prevalence and detection. Int J Geriatr Psychiatry.

[25] Pachana NA, Byrne GJA, Siddle H (2007). Development and validation of the geriatric anxiety inventory. Int Psychogeriatr.

[26] Antony M, Bieling PJ, Cox BJ (1998). Psychometric properties of the 42-item and 21-item versions of the depression anxiety stress scales in clinical groups and a community sample. Psychol Assess.

[27] Logsdon RG, Gibbons LE, McCurry SM, Teri L, Albert S, Logsdon RG (2000). Quality of life in Alzheimer’s disease: patient and caregiver reports. Assessing quality of life in Alzheimer’s disease.

[28] Edelman P, Fulton BR, Kuhn D, Chang C-H (2005). A comparison of three methods of measuring dementia-specific quality of life: perspectives of residents, staff, and observers. Gerontologist.

[29] Prager E (1986). Components of personal adjustment of long distance elderly movers. Gerontologist.

[30] Lawton MP (1971). Functional assessment of elderly people. J Am Geriatr Soc.

[31] Gagné M (2003). The role of autonomy support and autonomy orientation in prosocial behavior engagement. Motiv Emot.

[32] Ware JE, Snow KK, Kosinski M, Gandek B (1989). SF-36 health survey: manual and interpretation guide.

[33] Lemke S, Moos RH (1987). Measuring the social climate of congregate residences for older people: sheltered care environment scale. Psychol Aging.

[34] Hox J (2010). Multilevel analysis: techniques and applications.

[35] White IR, Horton NJ, Carpenter J, Pocock SJ (2011). Strategy for intention to treat analysis in randomised trials with missing outcome data. Br Med J.

