# Supplementary material for: A cluster randomised trial of the program to enhance adjustment to residential living (PEARL): a novel psychological intervention to reduce depression in newly admitted aged care residents
Source: BMC Geriatr. 2020 Mar 12;20:98. doi: 10.1186/s12877-020-1492-5 (PMC7068981; doi:10.1186/s12877-020-1492-5)
Supplement: Supplementary file 1 — Additional file 1. Scales designed specifically for this study. List of items in three scales were designed specifically for this trial: Meaningful Activity in Residential Care, Importance of Basic Needs, View of Relocation Scale. [file 12877_2020_1492_MOESM1_ESM.docx]

**Supplement S1: Scales designed specifically for this study**

**Meaningful Activity in Residential Care**

**Instructions:** For the following section, think about the activities you are involved in throughout your day. Please indicate how much you agree or disagree with each statement.

|  | Strongly disagree | Disagree | Agree | Strongly agree |
| --- | --- | --- | --- | --- |
| 1. I have a hobby or interest that I enjoy. | 1 | 2 | 3 | 4 |
| 1. I engage in daily activities that make life worthwhile. | 1 | 2 | 3 | 4 |
| 1. Social events don’t interest me much.* | 1 | 2 | 3 | 4 |
| 1. I look forward to visits from family or friends. | 1 | 2 | 3 | 4 |
| 1. The things I do day to day are fulfilling. | 1 | 2 | 3 | 4 |
| 1. There are not many things that interest me these days.* | 1 | 2 | 3 | 4 |
| 1. I don’t find many things to do here that are meaningful to me.* | 1 | 2 | 3 | 4 |
| 1. I feel bored much of the time.* | 1 | 2 | 3 | 4 |
| 1. I have lost most of my interests.* | 1 | 2 | 3 | 4 |

*Reverse scored items: 3, 6, 7, 8, 9

**Importance of Basic Needs**

**Instructions:** Please indicate how important each statement is for you.

| *How important is it…* | not important | somewhat important | very important |
| --- | --- | --- | --- |
| 1. …for you to decide for yourself how to live your life? | 0 | 1 | 2 |
| 1. …that you get along well with other people in your day to day life? | 0 | 1 | 2 |
| 1. …to you to have lots of social contacts? | 0 | 1 | 2 |
| 1. …to you to feel that there are things you are good at? | 0 | 1 | 2 |
| 1. …for you to be free to express your own ideas and opinions? | 0 | 1 | 2 |
| 1. …for you to have people in your life who care about you? | 0 | 1 | 2 |
| 1. …to be able to decide for yourself how to do things day to day? [for example, what clothes to wear, when to shower, what to eat] | 0 | 1 | 2 |
| 1. …to be close to many people? | 0 | 1 | 2 |
| 1. …to feel a sense of accomplishment in your daily life? | 0 | 1 | 2 |
| 1. …for other people to take your feelings into consideration? [e.g., your family or staff] | 0 | 1 | 2 |
| 1. …to have other people like you? | 0 | 1 | 2 |
| 1. …to have a chance to show how capable you are at things? | 0 | 1 | 2 |
| 1. …that other people are friendly towards you? | 0 | 1 | 2 |
| 1. …for you to do the things you can still do yourself (like washing, combing your hair, shaving) for as long as possible? | 0 | 1 | 2 |
| 1. …to like the people you interact with? | 0 | 1 | 2 |

Importance of autonomy: Items 1, 5, 7, 10

Important of relatedness: Items 2, 3, 6, 8, 11, 13, 15

Importance of competence: Items 4, 9, 12, 14

**View of Relocation Scale**

**Instructions:** For the following section, think about how you felt when you first arrived here about moving to this aged care home. Please indicate whether you mostly agree or disagree with each statement.

|  | Disagree | Agree |
| --- | --- | --- |
| 1. I felt that moving to a residential facility was the right decision for me. | 0 | 1 |
| 1. I was able to choose whether or not to move here. | 0 | 1 |
| 1. At this stage of my life, moving to a residential facility was appropriate. | 0 | 1 |
| 1. I was free to plan for the move as I wanted. | 0 | 1 |
| 1. Overall, I thought my needs would be best met in a residential facility. | 0 | 1 |
| 1. I felt pressured to move here by other people, such as my family, friends, or doctor.* | 0 | 1 |
| 1. I felt I needed the level of care provided by an aged care facility. | 0 | 1 |
| 1. I didn’t get much of a say in organising the move.* | 0 | 1 |
| 1. I felt unable to care for myself at home. | 0 | 1 |
| 1. The decision to move here was mostly mine to make. | 0 | 1 |
| 1. I could have continued to live at home for longer.* | 0 | 1 |
| 1. Other people did most of the day-to-day planning around the move.* | 0 | 1 |

Control in the decision to relocate: Items 2, 4, 6 (reverse scored*), 8 (reverse scored*), 10, 12 (reverse scored*)

The relocation was warranted: 1, 3, 5, 7, 9, 11 (reverse scored*)
